# Long-term patient experience with online MR-guided radiotherapy: adaptive versus non-adaptive workflow

**DOI:** 10.3389/fonc.2026.1700649

**Published:** 2026-02-03

**Authors:** Fabian Weykamp, Charlotte Herder-Wagner, Sebastian Regnery, Jakob Liermann, Eva Meixner, Philipp Hoegen-Saßmannshausen, Laila König, Kristin Lang, C. Katharina Renkamp, Carolin Rippke, Sebastian Klüter, Jürgen Debus, Juliane Hörner-Rieber

**Affiliations:** 1 Department of Radiation Oncology, Heidelberg University Hospital, Heidelberg, Germany; 2 Heidelberg Institute of Radiation Oncology (HIRO), Heidelberg, Germany; 3 National Center for Tumor Diseases (NCT), Heidelberg, Germany; 4 Clinical Cooperation Unit Radiation Oncology, German Cancer Research Center (DKFZ), Heidelberg, Germany; 5 Heidelberg Ion-Beam Therapy Center (HIT), Department of Radiation Oncology, Heidelberg University Hospital, Heidelberg, Germany; 6 German Cancer Consortium (DKTK), Partner Site Heidelberg, Heidelberg, Germany; 7 Department of Radiation Oncology, Duesseldorf University Hospital, Duesseldorf, Germany

**Keywords:** adaptive radiotherapy, local control (LC), MR-guided, patient-reported outcomes, stereotactic body radiotherapy (SBRT)

## Abstract

**Purpose/objective:**

Magnetic resonance–linear accelerator (MR-Linac) systems enable high-precision radiotherapy through real-time MR guidance and daily online adaptive treatment planning. While online adaptation offers substantial dosimetric advantages, it extends treatment session durations on an already resource-intensive platform. This study aimed to evaluate patient-reported outcome measures (PROMs) and long-term toxicity profiles associated with MR-guided radiotherapy, with a particular focus on the impact of online adaptive workflows.

**Materials and methods:**

This subgroup analysis of an ongoing prospective observational study comprises patients treated with the MRIdian Linac at the Department of Radiation Oncology at Heidelberg University Hospital between January 2019 and May 2021. Online plan adaptation was implemented in February 2020. A custom-designed in-house questionnaire (PRO-Q) was employed to assess patient experience with MR-guided treatment. Toxicity was classified according to the Common Terminology Criteria for Adverse Events (CTCAE v. 5.0).

**Results:**

A total of 231 patients were included, comprising 130 non-adaptive and 101 adaptive treatments across 286 target volumes. Baseline patient characteristics, prior systemic therapy, and median planning target volumes (36.4 mL *vs*. 35.3 mL) were comparable between groups. Adaptive treatment was associated with significantly prolonged session durations (median 71 minutes *vs*. 36 minutes; p<0.01). During adaptive treatment, patients reported significantly higher discomfort in domains related to treatment duration, immobility, and sensory perceptions (e.g., tingling) as per PRO-Q responses. No statistically significant differences in overall toxicity were observed. However, patients undergoing adaptive therapy exhibited a faster return to baseline status post-treatment (6–8 weeks *vs*. 6–12 months).

**Conclusion:**

Online plan adaptation at the MR-Linac increased treatment times and was associated with less favorable short-term patient-reported outcomes, yet it was delivered safely without compromising toxicity or oncologic outcomes. These results support adaptive MR-guided radiotherapy as a feasible and technically promising approach, while highlighting the need for further studies with validated PROMs and cost-benefit analyses to define its clinical value.

## 1 Background and purpose

Modern radiotherapy is increasingly benefiting from the growing possibilities of computer technology and the wider availability of sophisticated imaging techniques. It is now technically possible to modify a radiation treatment plan while the patient is still on the treatment couch, and MRI image guidance can be integrated into this process (1). Through its superior soft tissue contrast, online MR-guided radiotherapy allows for a more precise definition of tumor tissue and organs at risk (OARs). Moreover, with online treatment plan adaptation, changes in organ at risk (OAR) or tumor anatomy (e.g., bladder filling or tumor regression) can be accounted for on a daily basis (2–4). However, this adaptability comes at the cost of extended treatment times, imposing logistical and physical burdens on both patients and radiation oncology teams (5). The MR-Linac, as a hybrid device, is capable of both online MR guidance and online treatment plan adaptation. There is broad evidence for a dosimetric superiority of online MR-guided adaptive radiotherapy; however, there is currently limited robust randomized data available to demonstrate a translation into superior clinical outcomes (6–11). Dosimetric data for evaluating online adaptive radiotherapy are readily obtainable, as both adapted and non-adapted treatment plans are available for analysis without the need for a patient control group.

In our prior work, we showed that, among others, online adaptive MR-guided radiotherapy provides consistent dosimetric advantages across multiple tumor sites, including lung, liver, adrenal, lymphatic, and prostate tumors. We demonstrated that daily adaptive replanning enables reduced safety margins and improves dose conformity while maintaining or safely escalating ablative dose levels. Our results showed that up to 32.9% of non-adapted plans exhibited OAR constraint violations, and up to 99.4% demonstrated planning target volume coverage violations, both of which could be resolved through plan adaptation. The dosimetric benefit was most pronounced for tumors in high-risk or anatomically complex locations characterized by significant interfractional anatomical variation and motion. Collectively, these findings establish a robust dosimetric foundation supporting the clinical outcomes reported in the present study (11–17).

As far as workflow descriptions on the MR-Linac are concerned, studies on several hundred patients are now available (18, 19). Even without treatment adaptation, MR-guided stereotactic radiotherapy of liver metastases, for example, has a median session duration of 39 minutes (20). However, patient-reported outcome measures (PROMs) are especially important for resource-intensive treatment techniques such as the MR-Linac, since it requires a high degree of patient compliance (21). Preliminary international PROMs of MR-Linac treatment and early toxicity rates were positive (22–26). However, comprehensive assessments of long-term toxicity are sparse, and, to date, no direct comparison of PROMs between adaptive and non-adaptive MR-Linac treatments has been reported. In our previous work, we were able to demonstrate the acceptance and good clinical tolerability of MR-guided radiotherapy. However, that work was conducted at a time when adaptive radiotherapy was not yet established in our center (10, 20). We sought to evaluate the PROMs and long-term toxicity associated with MR-guided radiotherapy, with a particular focus on the impact of online adaptive workflows.

## 2 Methods

This is an analysis from a prospective observational trial comprising all cancer patients referred to our institution for online MR-guided radiotherapy. Patients were treated with MR-guided radiotherapy at the MRIdian Linac (ViewRay Inc., Mountain View, CA) at the Department of Radiation Oncology at Heidelberg University Hospital between January 2019 and May 2021. Radiotherapy dose and fractionation schemes were prescribed in accordance with tumor-specific standard operating procedures (SOPs) established at our institution. SBRT was defined according to the guideline of the working group “Stereotactic Radiotherapy” of the German Society of Radiation Oncology (single fraction dose ≥ 4Gy and number of fractions ≤12) (27). The implementation of online treatment plan adaptation commenced in February 2020. Online plan adaptation was implemented to account for anatomical changes, improve target volume coverage and enhance sparing of organs at risk. Since evidence on which anatomical treatment sites benefit most from adaptation was limited at the time, we recommended a broad inclusion framework. However, we subsequently discontinued treating patients with bone metastases on the MR-Linac, as no relevant anatomical changes were expected for this specific anatomical site. A subset of patients who received MR-guided radiotherapy for liver and lymph node metastases had previously been reported on in relation to dosimetric parameters and clinical outcomes. These patients (20 patients with liver metastases and 29 patients with lymph node metastases) were included in the present analysis with extended follow-up (10, 20). A comprehensive description of the MR-Linac treatment protocols, including patient selection, simulation, planning, and delivery workflows, has been published previously (28).

The simulation MR images, which served as the primary treatment planning modality, were acquired either during breath-hold in deep inspiration or during free breathing using the TrueFISP sequence. Different in-plane resolutions were used for the 3D simulation MRI (either 1.5 × 1.5mm² or 1.6 × 1.6mm² and slice thickness of 3mm with different fields of view). Sagittal-plane cine MRI images obtained with the TrueFISP sequence were utilized to assess movement. No contrast agent was administered. CT simulation scans were additionally obtained (with or without contrast agent) with the same specifications as the MR simulation and then deformably registered. Clinical target volume margins ranged from 1 mm for pelvic lymph nodes to 5 mm for liver metastases, depending on the anatomical region. In general, a 3 mm margin was added as the planning target volume. Following this, step-and-shoot IMRT treatment plans were generated using the integrated MRIdian planning system. The Monte Carlo dose calculation accounted for the static magnetic field.

To systematically evaluate patient experience with MR-Linac-based radiotherapy, we used an in-house-designed patient-reported outcome questionnaire (PRO-Q) (28). This PRO-Q dates back to the early days of implementing MR-Linac therapy at our center. It is a non-validated tool designed by us for internal quality control. The instrument employs a 5-point Likert scale, with scores ranging from 1 (very positive experience) to 5 (very negative experience). To complement the patient-reported data and provide additional insight into treatment complexity, MR-Linac staff were also asked to assess each patient’s treatment course using a 10-point scale, where 1 indicated minimal difficulty and 10 represented a level of effort approaching unacceptability. The resulting scores were evaluated descriptively for each patient.

Before the first and last treatment session, as well as at first follow-up, each patient was specifically assessed for the presence of fatigue, nausea, vomiting, diarrhea, constipation, dyspnea, cough, skin disorder, and pain. Follow-up started 6–8 weeks after the last treatment day together with a clinical examination and a contrast-enhanced MRI or CT scan if indicated according to the respective institutional guidelines. Further follow-up was not part of the prospective study. Hence, assessment at later follow-up points was performed retrospectively. The Response Evaluation Criteria in Solid Tumors (RECIST 1.1) and the Common Terminology Criteria for Adverse Events (CTCAE v. 5.0) were used for tumor response and toxicity assessment, respectively.

Patient and treatment characteristics were analyzed descriptively (IBM SPSS Version 28.0). Group comparisons were performed using the chi-square test or the Mann–Whitney U test. Multiple responses were taken into account to analyze toxicity. When first- and second-grade toxicity were reported simultaneously, the higher grade was counted for clarity. However, the severity of toxicity was documented at each grade to obtain a complete picture. Toxicity was compared using the chi-square test. To assess PROMs, the Wilcoxon signed-rank test was used to compare patient experiences after the first and last radiation fraction. The Mann–Whitney U test was used to compare patient groups with and without online adaptation. Local control (LC) and overall survival (OS) were estimated starting from the first day of the irradiation. LC was calculated based on each irradiated lesion. OS was calculated per patient. LC and OS were estimated using the Kaplan–Meier method, and significance was tested using the log-rank method. A significance level of α=5% was utilized. The MR-Linac observational study was approved by the Ethics committee of the University Hospital Heidelberg (S-543/2018, S-862/2019).

## 3 Results

**Figure 1:**
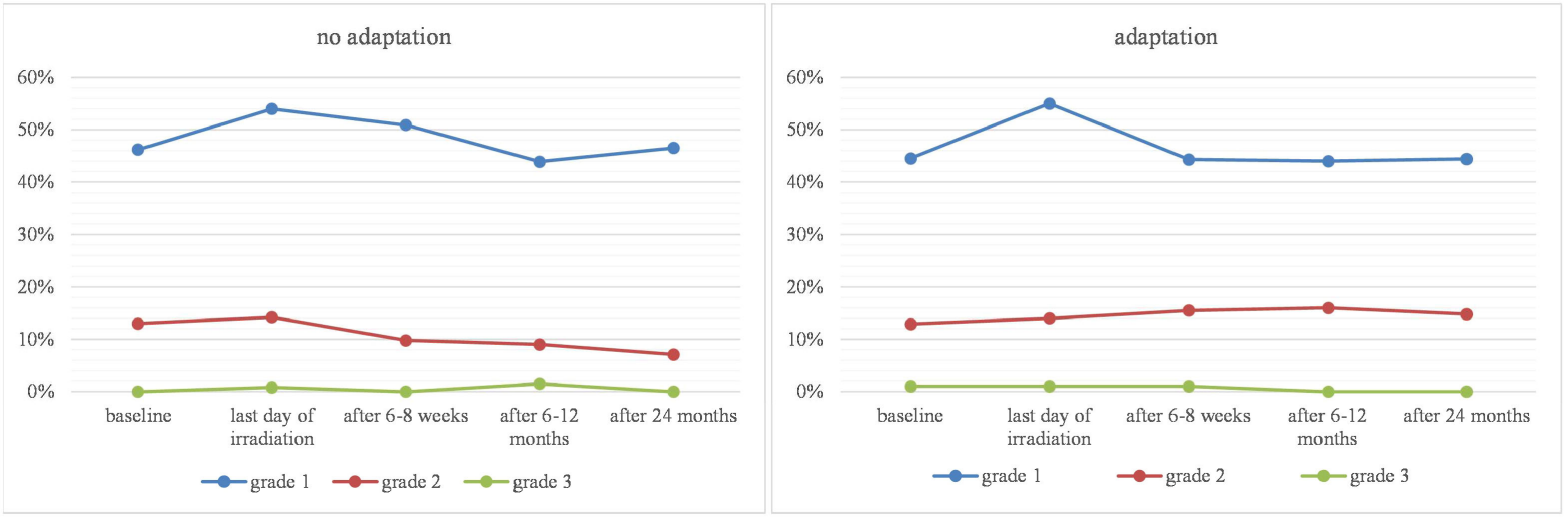
Comparison of long-term toxicity (no adaption *vs*. adaption).

Between January 2019 and May 2021, 231 patients were treated at our MR-Linac (n=130 before and n=101 after the implementation of adaptive radiotherapy 02/2020). In total, n=286 tumor lesions were targeted and n=2020 fractions were applied. Both treatment groups (non-adaptive/adaptive) were similar in terms of age (65 years, range: 28–84 years *vs*. 66 years; range: 19–89 years), sex, body mass index, performance score, and comorbidity index (**Table 1**). Median planning target volumes (36.4 mL *vs*. 35.3 mL) were comparable between both groups. The predominant primary tumors were lung cancer, prostate cancer, and breast cancer, which did not significantly differ between the two treatment groups (**Table 2**). The most prevalent targets were lymph node and liver metastases (**Table 3**). Most patients did not receive systemic therapy four weeks before or after MR-guided radiotherapy. **Table 4** provides an overview of the systemic therapy that was administered. The treatment characteristics (**Table 5**) were comparable between the two groups, but the median treatment duration increased from 36 minutes to 71 minutes after the implementation of adaptive radiotherapy. Toxicity was low in general, with no grade 4 or higher toxicity at all and only one case of grade 3 toxicity at any time. There was no significant difference between the treatment groups (**Table 6**), but descriptive analysis revealed that grade 1 toxicity already returned to baseline at first follow-up in the adaptive treatment group. **Figure 1** depicts the toxicity over time. **Table 7** provides an overview of the symptoms reported. Fatigue was by far the most common symptom and was already present at baseline in 28% of the patients.

**Table 1:** Patient characteristics.

Patient characteristics	No adaptation (n=130)	Adaptation (n=101)	p
Mean age	65 years	66 years	0.374
	(range 28–84 years)	(range 19–89 years)	
Female/male	77/53	67/34	0.269
	(59.2%/40.8%)	(66.3%/33.7%)	
Mean body mass index	25.3 kg/m²	25.8 kg/m²	0.852
	(range 17.3 – 42.3 kg/m²)	(range 17.5 – 40.4 kg/m²)	
Mean Karnofsky score	90%	90%	0.121
	(range 70 - 100%)	(range 50 - 100%)	
Mean Charlson comorbidity index	7 points	8 points	0.822
	(range 3–13 points)	(range 2–13 points)	
Curative/palliative treatment intent	123/7	100/1	0.070
	(94.6%/5.4%)	(99.0%/1.0%)	

**Table 2:** Primary tumor.

No adaption (n = 130)			Adaption (n = 101)			p
Lung cancer	30	23.1%	Lung cancer	24	23.7%	0.903
Prostate cancer	28	21.5%	Prostate cancer	25	24.7%	0.564
Breast cancer	15	11.6%	Breast cancer	10	9.9%	0.691
Colorectal cancer	13	10.0%	Colorectal cancer	11	10.9%	0.826
Melanoma	8	6.2%	Melanoma	5	5.0%	0.694
Kidney cancer	6	4.6%	Kidney cancer	3	3.0%	0.522
Other	30	23.0%	Other	23	22.8%	0.195

**Table 3:** Localization of target volumes.

No adaption (n = 165)			Adaption (n = 121)			p
Lymph node	47	28.5%	Lymph node	38	31.4%	0.593
Liver	32	19.4%	Liver	30	24.8%	0.274
Lung	32	19.4%	Lung	27	22.3%	0.547
Bone	23	13.9%	Bone	0	0.0%	**<0.001**
Adrenal gland	9	5.5%	Adrenal gland	10	8.3%	0.346
Soft tissue	8	4.8%	Soft tissue	2	1.7%	0.146
Prostate	1	0.6%	Prostate	5	4.1%	**0.040**
Other	13	7.9%	Other	9	7.4%	0.890

Bold values indicate statistically significant.

**Table 4:** Systemic therapy 4 weeks before and after irradiation.

Time point/systemic therapy		No adaption (N = 130)		Adaption (N = 101)		p
4 weeks before irradiation	Chemotherapy	15	11.5%	6	5.9%	0.142
	Anti-hormonal therapy	14	10.8%	11	10.9%	0.976
	Immunotherapy	11	8.5%	5	5.0%	0.217
	Targeted therapy	10	7.7%	8	7.9%	0.949
4 weeks after irradiation	Chemotherapy	10	7.7%	6	5.9%	0.603
	Anti-hormonal therapy	15	11.5%	11	10.9%	0.877
	Immunotherapy	14	10.8%	5	5.0%	0.110
	Targeted therapy	8	6.2%	9	8.9%	0.426

Bold values indicate statistically significant.

**Table 5:** Treatment characteristics.

Treatment characteristics		No adaption (n = 165)		Adaption (n = 121)		p
Targets per treatment series	n = 1	156	94.6%	104	86.0%	0.050
	n = 2	8	4.8%	15	12.4%	
	n = 3	1	0.6%	2	1.6%	
Total number of treatment series	1	105	80.8%	86	85.1%	0.386
	2	17	13.1%	12	11.9%	
	3	6	4.6%	2	2.0%	
	4	2	1.5%	0	0%	
	5	0	0%	1	1.0%	
		median	range	median	range	
Gross tumor volume		10.7 mL	0.1 - 848.3 mL	10.1 mL	0.2 – 196.1 mL	0.461
Clinical target volume		19.9 mL	0.4 - 1099.5 mL	24.5 mL	0.4 – 307.1 mL	0.530
Planning target volume		36.4 mL	2.6 - 1253.0 mL	35.3 mL	1.5 – 376.5 mL	0.159
Total dose		39.0 Gy	4.0 – 66.0 Gy	50.0 Gy	25.0 – 65.0 Gy	**0.005**
Single dose		7.5 Gy	1.8 – 15.0 Gy	7.5 Gy	2.67 – 15.0 Gy	0.166
Fractions		6	2 - 33	6	3 - 15	0.536
Monitor units per fraction		1747.1	366.9 - 6309.7	1700.1	344.7 – 5247.0	0.641
Session duration (“on table”)		36.0 min	11.0-93.0 min	70.9 min	22.7-100.4 min	**<0.001**

**Table 6:** Comparison of toxicity (CTCAE).

Time point	No adaptation								Adaptation								p
	No toxicity		Grade 1		Grade 2		Grade 3		No toxicity		Grade 1		Grade 2		Grade 3		
Baseline	53	40.8%	60	46.2%	17	13.0%	0	0%	42	41.6%	45	44.5%	13	12.9%	1	1.0%	0.722
Last irradiation day	39	31.0%	68	54.0%	18	14.2%	1	0.8%	30	30.0%	55	55.0%	14	14.0%	1	1.0%	0.732
After 6–8 weeks	44	39.3%	57	50.9%	11	9.8%	0	0%	38	39.2%	43	44.3%	15	15.5%	1	1.0%	0.399
After 6–12 months	31	46.2%	29	43.3%	6	9.0%	1	1.5%	20	40.0%	22	44.0%	8	16.0%	0	0%	0.645
After 24–36 months	20	46.5%	20	46.5%	3	7.0%	0	0%	11	40.8%	12	44.4%	4	14.8%	0	0%	0.659

Available data. Baseline: n = 130 no adaption/n = 101 adaptation; last irradiation day: n = 125/n = 99 (1 death); after 6–8 weeks: n = 112/97 (1 death); after 12 months: n = 67 (16 deaths)/n = 50 (15 deaths); after 36 months: n = 43 (43 deaths)/n = 27 (34 deaths).

**Table 7:** Specification of toxicity.

Time point	Symptom	No adaptation						Adaptation						statistical
		Grade 1		Grade 2		Grade 3		Grade 1		Grade 2		Grade 3		
Baseline	Fatigue	37	28.5%	5	3.8%	0	–	28	27.7%	2	2.0%	0	–	0.698
	Nausea/Vomiting	2	1.5%	1	0.8%	0	–	8	7.9%	0	–	1	1%	0.053
	Dysphagia	4	3.1%	0	–	0	–	3	3.0%	0	–	0	–	0.963
	Dyspepsia	10	7.7%	0	–	0	–	5	5.0%	1	1.0%	0	–	0.375
	Diarrhea	5	3.8%	1	0.8%	0	–	5	5.0%	2	2.0%	0	–	0.659
	Constipation	6	4.6%	2	1.5%	0	–	6	5.9%	0	–	0	–	0.418
	Flatulence	11	8.5%	0	–	0	–	9	8.9%	0	–	0	–	0.904
	Cystitis	2	1.5%	0	–	0	–	5	5.0%	0	–	0	–	0.133
	Dyspnea	14	10.8%	3	2.3%	0	–	12	11.9%	4	4.0%	0	–	0.731
	Cough	11	8.5%	1	0.8%	0	–	9	8.9%	1	1.0%	0	–	0.976
	Radiation dermatitis	2	1.5%	0	–	0	–	3	3.0%	2	2.0%	0	–	0.204
	Pain	25	19.2%	8	6.2%	0	–	12	11.9%	5	5.0%	0	–	0.273
	Other	8	6.2%	0	–	0	–	11	10.9%	3	3.0%	0	–	0.098
Last day of irradiation	Fatigue	53	42.4%	13	10.4%	0	–	49	49.0%	8	8.0%	0	–	0.657
	Nausea/Vomiting	19	15.2%	4	3.2%	0	–	17	17.0%	1	1.0%	0	–	0.354
	Dysphagia	1	0.8%	0	–	0	–	5	5.0%	1	1.0%	1	1%	0.072
	Dyspepsia	4	3.2%	0	–	0	–	9	9.0%	1	1.0%	0	–	0.082
	Diarrhea	6	4.8%	1	0.8%	0	–	8	8.0%	0	–	0	–	0.398
	Constipation	3	2.4%	0	–	0	–	2	2.0%	0	–	0	–	0.865
	Flatulence	7	5.6%	0	–	0	–	3	3.0%	0	–	0	–	0.614
	Cystitis	0	–	0	–	0	–	4	4.0%	0	–	0	–	**0.022**
	Dyspnea	4	3.2%	2	1.6%	0	–	11	11.0%	2	2.0%	0	–	0.065
	Cough	9	7.2%	1	0.8%	0	–	8	8.0%	0	–	0	–	0.584
	Radiation dermatitis	1	0.8%	0	–	0	–	5	5.0%	0	–	0	–	0.098
	Pain	16	12.8%	1	0.8%	0	–	11	11.0%	3	3.0%	0	–	0.390
	Other	9	7.2%	2	1.6%	1	0.8%	4	4.0%	1	1.0%	0	–	0.674
After 6–8 weeks	Fatigue	32	28.6%	2	1.8%	0	–	35	36.1%	4	4.1%	0	–	0.266
	Nausea/Vomiting	5	4.5%	0	–	0	–	2	2.1%	3	3.1%	1	1%	0.137
	Dysphagia	1	0.9%	1	0.9%	0	–	5	5.2%	1	1.0%	0	–	0.203
	Dyspepsia	0	–	0	–	0	–	2	2.1%	2	2.1%	0	–	0.095
	Diarrhea	5	4.5%	0	–	0	–	4	4.1%	0	–	0	–	0.902
	Constipation	7	6.3%	0	–	0	–	9	9.3%	0	–	0	–	0.413
	Flatulence	3	2.7%	0	–	0	–	2	2.1%	0	–	0	–	0.770
	Cystitis	2	1.8%	0	–	0	–	4	4.1%	0	–	0	–	0.314
	Dyspnea	13	11.6%	3	2.7%	0	–	8	8.2%	6	6.2%	0	–	0.403
	Cough	19	17.0%	0	–	0	–	11	11.3%	0	–	0	–	0.246
	Radiation dermatitis	3	2.7%	0	–	0	–	1	1.0%	0	–	0	–	0.385
	Pain	13	11.6%	2	1.8%	0	–	12	12.4%	3	3.1%	0	–	0.975
	Other	15	13.4%	1	0.9%	0	–	10	10.3%	2	2.1%	0	–	0.900

Bold values indicate statistically significant.

**Figure 2:**
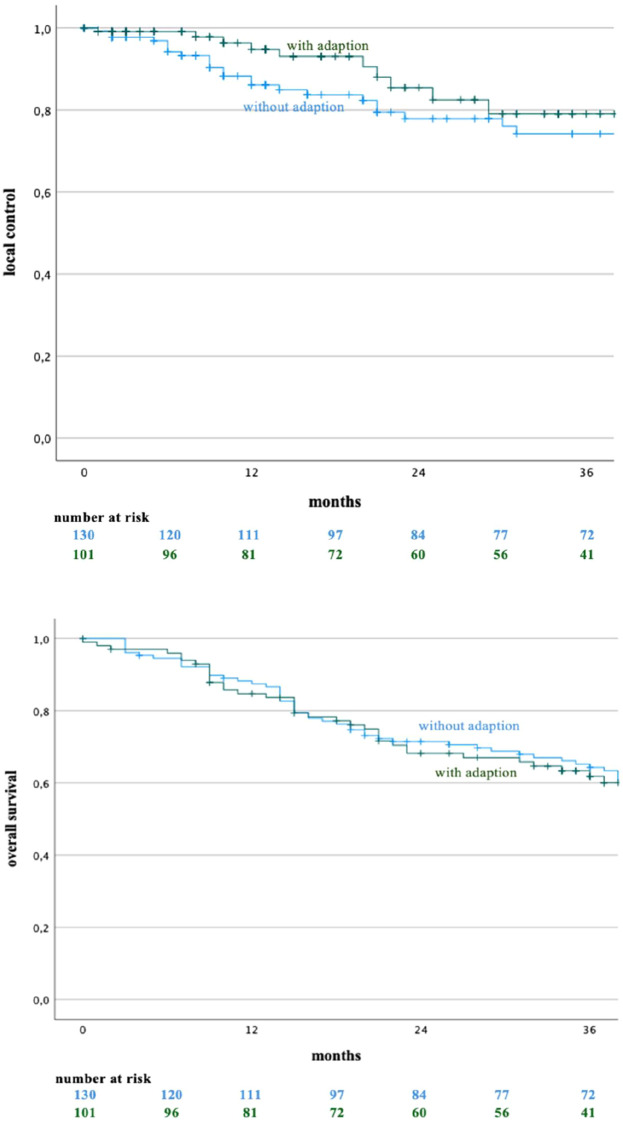
Local control (p=0.544) and overall survival (p = 0.875) compared between no adaption and adaption.

Three items within the PRO-Q were rated significantly less favorably by patients in the adaptive treatment group (**Table 8**). Specifically, patients in the adaptive cohort reported greater dissatisfaction with the overall treatment duration, the requirement to remain immobile during the procedure, and the occurrence of tingling sensations in the extremities, with mean scores of 3.3 *vs*. 2.5, 2.6 *vs*. 2.2, and 2.2 *vs*. 1.8, respectively. In addition, treatment complexity, as assessed by MR-Linac personnel, was rated significantly higher in the adaptive group, with a mean score of 4.8 compared to 4.1. The median follow-up across the cohort was 35 months. In an exploratory analysis, treatment duration showed no additional association with patient-reported experience or acute toxicity beyond the effects of adaptive versus non-adaptive workflows. No statistically significant differences in local control (LC) or overall survival (OS) were observed between the adaptive and non-adaptive groups (**Figure 2**).

**Table 8:** Results of the patient-reported outcome questionnaire (positions 1-17) and the staff questionnaire (position 18).

Item	No adaptation (n = 119)				Adaptation (n = 94)				*p*
	Mean	*p*	Median	Range	Mean	*p*	Median	Range	
1. Overall treatment experience	1.5	*0.415*	1	1-5	1.8	*0.211*	2	1-5	*0.158*
2. Information provided by the staff	1.2	*0.819*	1	1-5	1.4	*0.796*	1	1-4	*0.283*
3. Friendliness of the staff	1.1	*0.083*	1	1-5	1.1	*0.414*	1	1-5	*0.061*
4. Duration of the treatment	2.5	*0.947*	2	1-5	3.3	***0.012***	3	1-5	***<0.001***
5. Size of the MRI bore	2.1	*0.741*	2	1-5	2.3	*1.000*	2	1-5	*0.206*
6. Positioning during radiotherapy	2.3	*0.520*	2	1-5	2.6	*0.906*	3	1-5	*0.059*
7. Having to lie still	2.2	*0.331*	2	1-5	2.6	*0.882*	3	1-5	***0.011***
8. Noise in the MR-Linac	2.3	*0.272*	2	1-5	2.4	*0.074*	2	1-5	*0.532*
9. Temperature in the MR-Linac	2.6	*0.701*	3	1-5	2.6	*0.987*	3	1-5	*0.498*
10. Local temperature of body parts	2.6	*0.961*	3	1-5	2.6	*0.977*	3	1-4	*0.941*
11. Tingling sensations in fingers and toes	1.8	*0.192*	1	1-5	2.2	*0.969*	2	1-5	***0.021***
12. Breathing instructions	1.3	*0.138*	1	1-5	1.4	*0.125*	1	1-5	*0.914*
13. Breath holding	1.6	*1.000*	1	1-5	1.6	*0.726*	1	1-5	*0.909*
14. Anxiousness during treatment session	1.6	*0.766*	1	1-5	1.8	*0.358*	1	1-4	*0.061*
15. Difficulty to hold the target with one’s own breath	1.5	*0.796*	1	1-4	1.4	*0.317*	1	1-4	*0.509*
16. Ability to watch one’s own treatment via monitor	1.3	*0.285*	1	1-4	1.2	*0.317*	1	1-3	*0.482*
17. Feeling of having active control over the treatment duration	1.3	*0.763*	1	1-4	1.1	*0.157*	1	1-2	*0.585*
18. Treatment complexity from the perspective of the staff	4.1	*0.113*	4	1-10	4.8	***0.032***	5	1-10	***0.004***

The outcome reports were reviewed for significant differences both between the first and last session and between the different patient groups. Bold values indicate statistically significant.

## 4 Discussion

This analysis presents patient-reported outcomes and long-term toxicity data from a cohort of 231 cancer patients treated with MR-guided radiotherapy on an MR-Linac system between January 2019 and May 2021. To our knowledge, this represents the largest single-institution series to date that specifically evaluates these parameters in the MR-Linac setting. The mid-study implementation of online adaptive radiotherapy in February 2020 afforded a unique opportunity to compare adaptive and non-adaptive workflows within a consistent institutional framework. Nearly half of our patients were treated for liver or lymph node metastases - disease sites particularly well suited for MR guidance given its superior soft-tissue contrast (29, 30).

The primary objective of this study was to evaluate long-term patient experience and acceptance of online MR-guided radiotherapy workflows, comparing adaptive and non-adaptive approaches, rather than to perform a definitive clinical efficacy comparison. Although baseline patient characteristics, prior systemic therapy, and median planning target volumes were comparable between groups, the non-adaptive MR-guided cohort reflects treatments delivered before routine implementation of online adaptation and therefore represents a heterogeneous group influenced by temporal, technical, and workflow-related factors. Moreover, adaptive treatments enabled delivery of significantly higher doses, further limiting the feasibility of a direct, uniform clinical comparison. Consequently, clinical outcome and toxicity data are reported descriptively and should be interpreted with caution, as acknowledged.

Consistent with other early clinical reports, overall treatment experience in our cohort was rated favorably (22, 23, 31, 32). However, the transition to online adaptation resulted in a doubling of median treatment session duration (36 *vs*. 71 minutes), which was associated with less favorable patient-reported outcomes in some domains. This effect is plausibly linked to the longer immobilization time and cooler treatment environment. Importantly, levels of anxiousness and overall treatment satisfaction did not deteriorate, suggesting that patients generally tolerated the increased burden. Our institutional experience also demonstrates that simple supportive measures, such as providing additional blankets, can improve patient comfort. Nevertheless, these findings highlight the need to balance technical precision against treatment burden and to prioritize workflow optimization and patient comfort when implementing adaptation (6–11, 15).

Treatment-related toxicity in our cohort was low, with no grade ≥4 events and durable safety confirmed after a median follow-up of 35 months - among the longest reported in MR-Linac literature (33–35). We found no significant differences in toxicity between adaptive and non-adaptive treatments. Interestingly, patients in the adaptive group tended to return to baseline symptoms earlier, although this trend did not reach statistical significance. Overall, our results indicate that adaptive therapy can be delivered safely without compromising oncologic outcomes, but measurable advantages in toxicity, local control, or survival were not observed in this retrospective comparison.

At first glance, adaptive radiotherapy may therefore appear less desirable from a patient perspective, as it prolongs treatment sessions and is associated with less favorable short-term patient-reported outcomes, without showing clinical differences in toxicity or survival. However, our study has several important limitations. First, the primary outcome instrument (PRO-Q) was developed internally and has not undergone formal external validation. Although it has proven useful for internal quality assurance, this limits the generalizability of our results. Second, the adaptive and non-adaptive groups were not concurrently assigned but defined by the timeline of implementation, introducing both temporal and selection bias. Third, structured prospective toxicity monitoring ended after the first follow-up, with long-term data collected retrospectively, raising the possibility of underreporting. These methodological constraints limit the strength of causal inference.

Despite these limitations, our findings are consistent with international experiences reporting favorable patient tolerance and low toxicity in MR-guided therapy (22). Reports from Tübingen, Amsterdam, Istanbul, Utrecht, and Toronto have similarly documented positive patient experiences, highlighting the global reproducibility of MR-Linac outcomes (23–26, 31, 32). Beyond patient experience, MR guidance itself provides independent advantages - including real-time tumor visualization, gating, and functional imaging- that are not contingent on adaptive workflows (36, 37). The MIRAGE trial, for example, demonstrated significantly reduced genitourinary and gastrointestinal toxicity and improved patient-reported bowel outcomes in prostate cancer patients treated with online MR guidance, despite not employing plan adaptation (38). This underscores that MR guidance alone confers important clinical benefits, and that the incremental contribution of adaptation must be carefully disentangled in future studies (7, 15, 39, 40).

From a technical perspective, the dosimetric advantages of online adaptive radiotherapy are well established and are likely to translate into clinical benefit over time, as was the case during the transition from 3D-CRT to IMRT (41–43). However, the increased time required for adaptive workflows currently narrows the patient population who can feasibly benefit (44). Ongoing developments in workflow automation, deformable image registration, and artificial intelligence are expected to improve efficiency and patient tolerance. Furthermore, CT-based online adaptive systems are emerging and offer faster session times. These systems can also be coupled with weekly offline MR guidance (MARS) (45–47). We are currently investigating such approaches prospectively within our AIM-C1 trial for cervical cancer (48). These evolving strategies highlight the importance of tailoring adaptive and MR-guided workflows to patient-specific needs and treatment contexts.

## 5 Conclusion

The MR-Linac platform enables advanced radiotherapy delivery by combining real-time MR guidance with online plan adaptation. In this large single-institution series with long-term follow-up, we confirmed low treatment-related toxicity and overall favorable patient-reported outcomes. Adaptive therapy was associated with longer treatment times and less favorable ratings in certain patient-reported domains. The clinical relevance of the observed PROMs differences remains uncertain, but our findings underscore the importance of optimizing workflow efficiency and patient comfort when applying adaptive techniques. Future studies should clarify the unique contribution of adaptation, independent of the broader benefits of MR guidance, through randomized trials, validated PROMs instruments, and cost-benefit evaluations. Until such evidence is available, adaptive MR-guided radiotherapy should be applied selectively and particularly in patients where anatomical changes or organ-at-risk proximity make plan adaptation most likely to improve the therapeutic ratio.

## Data Availability

The data presented in this study will be available on reasonable request.

## References

[1] Webster M Podgorsak A Li F Zhou Y Jung H Yoon J . New approaches in radiotherapy. Cancers. (2025) 17:1980. doi: 10.3390/cancers17121980, PMID: 40563630 PMC12190917

[2] Ugurluer G Mustafayev TZ Gungor G Atalar B Abacioglu U Sengoz M . Stereotactic MR-guided online adaptive radiation therapy (SMART) for the treatment of liver metastases in oligometastatic patients: initial clinical experience. Radiat Oncol J. (2021) 39:33. doi: 10.3857/roj.2020.00976, PMID: 33794572 PMC8024184

[3] Daamen LA de Mol van Otterloo SR van Goor IW Eijkelenkamp H Erickson BA Hall WA . Online adaptive MR-guided stereotactic radiotherapy for unresectable Malignancies in the upper abdomen using a 1.5 T MR-linac. Acta Oncol. (2022) 61:111–5. doi: 10.1080/0284186X.2021.2012593, PMID: 34879792

[4] Stanescu T Shessel A Carpino-Rocca C Taylor E Semeniuk O Li W . MRI-guided online adaptive stereotactic body radiation therapy of liver and pancreas tumors on an MR-linac system. Cancers. (2022) 14:716. doi: 10.3390/cancers14030716, PMID: 35158984 PMC8833602

[5] Werensteijn-Honingh AM Kroon PS Winkel D Aalbers EM van Asselen B Bol GH . Feasibility of stereotactic radiotherapy using a 1.5 T MR-linac: Multi-fraction treatment of pelvic lymph node oligometastases. Radiotherapy Oncol. (2019) 134:50–4. doi: 10.1016/j.radonc.2019.01.024, PMID: 31005224

[6] Padgett KR Simpson G Asher D Portelance L Bossart E Dogan N . Assessment of online adaptive MR-guided stereotactic body radiotherapy of liver cancers. Physica Medica. (2020) 77:54–63. doi: 10.1016/j.ejmp.2020.07.027, PMID: 32781388

[7] Mayinger M Ludwig R Christ SM Dal Bello R Ryu A Weitkamp N . Benefit of replanning in MR-guided online adaptive radiation therapy in the treatment of liver metastasis. Radiat Oncol. (2021) 16:84. doi: 10.1186/s13014-021-01813-6, PMID: 33947429 PMC8097956

[8] Rogowski P von Bestenbostel R Walter F Straub K Nierer L Kurz C . Feasibility and early clinical experience of online adaptive MR-guided radiotherapy of liver tumors. Cancers. (2021) 13:1523. doi: 10.3390/cancers13071523, PMID: 33810244 PMC8037065

[9] Nierer L Eze C da Silva Mendes V Braun J Thum P von Bestenbostel R . Dosimetric benefit of MR-guided online adaptive radiotherapy in different tumor entities: liver, lung, abdominal lymph nodes, pancreas and prostate. Radiat Oncol. (2022) 17:53. doi: 10.1186/s13014-022-02021-6, PMID: 35279185 PMC8917666

[10] Weykamp F Herder-Wagner C Regnery S Hoegen P Renkamp CK Liermann J . Stereotactic body radiotherapy of lymph node metastases under MR-guidance: First clinical results and patient-reported outcomes. Strahlentherapie und Onkologie. (2022) 198:56–65. doi: 10.1007/s00066-021-01834-w, PMID: 34468783 PMC8760210

[11] Regnery S Buchele C Piskorski L Weykamp F Held T Eichkorn T . SMART ablation of lymphatic oligometastases in the pelvis and abdomen: clinical and dosimetry outcomes. Radiotherapy Oncol. (2022) 168:106–12. doi: 10.1016/j.radonc.2022.01.038, PMID: 35121031

[12] Regnery S Buchele C Weykamp F Pohl M Hoegen P Eichkorn T . Adaptive MR-guided stereotactic radiotherapy is beneficial for ablative treatment of lung tumors in high-risk locations. Front Oncol. (2021) 11:757031. doi: 10.3389/fonc.2021.757031, PMID: 35087746 PMC8789303

[13] Hoegen-Saßmannshausen P Renkamp CK Lau HH Neugebauer D Niebuhr N Buchele C . Prospective planning comparison of magnetic resonance-guided vs. internal target volume-based stereotactic body radiotherapy of hepatic metastases - Which patients do really benefit from an MR-linac? Clin Trans Radiat Oncol. (2025) 52:100941. doi: 10.1016/j.ctro.2025.100941, PMID: 40124646 PMC11926716

[14] Hoegen P Katsigiannopulos E Buchele C Regnery S Weykamp F Sandrini E . Stereotactic magnetic resonance-guided online adaptive radiotherapy of adrenal metastases combines high ablative doses with optimized sparing of organs at risk. Clin Trans Radiat Oncol. (2023) 39:100567. doi: 10.1016/j.ctro.2022.100567, PMID: 36935853 PMC10014324

[15] Weykamp F Katsigiannopulos E Piskorski L Regnery S Hoegen P Ristau J . Dosimetric benefit of adaptive magnetic resonance-guided stereotactic body radiotherapy of liver metastases. Cancers. (2022) 14:6041. doi: 10.3390/cancers14246041, PMID: 36551527 PMC9775484

[16] Fink CA Buchele C Baumann L Liermann J Hoegen P Ristau J . Dosimetric benefit of online treatment plan adaptation in stereotactic ultrahypofractionated MR-guided radiotherapy for localized prostate cancer. Front Oncol. (2024) 14:1308406. doi: 10.3389/fonc.2024.1308406, PMID: 38425342 PMC10902126

[17] Buchele C Renkamp CK Regnery S Behnisch R Rippke C Schlüter F . Intrafraction organ movement in adaptive MR-guided radiotherapy of abdominal lesions - dosimetric impact and how to detect its extent in advance. Radiat Oncol (London England). (2024) 19:80. doi: 10.1186/s13014-024-02466-x, PMID: 38918828 PMC11202341

[18] Garcia Schüler HI Pavic M Mayinger M Weitkamp N Chamberlain M Reiner C . Operating procedures, risk management and challenges during implementation of adaptive and non-adaptive MR-guided radiotherapy: 1-year single-center experience. Radiat Oncol. (2021) 16:217. doi: 10.1186/s13014-021-01945-9, PMID: 34775998 PMC8591958

[19] Henke LE Contreras JA Green OL Cai B Kim H Roach MC . Magnetic resonance image-guided radiotherapy (MRIgRT): A 4.5-year clinical experience. Clin Oncol. (2018) 30:720–7. doi: 10.1016/j.clon.2018.08.010, PMID: 30197095 PMC6177300

[20] Weykamp F Hoegen P Klüter S Spindeldreier CK König L Seidensaal K . Magnetic resonance-guided stereotactic body radiotherapy of liver tumors: initial clinical experience and patient-reported outcomes. Front Oncol. (2021) 11:2103. doi: 10.3389/fonc.2021.610637, PMID: 34178616 PMC8219972

[21] Weldring T Smith SM . Article commentary: patient-reported outcomes (PROs) and patient-reported outcome measures (PROMs). Health Serv Insights. (2013) 6:HSI. S11093. doi: 10.4137/HSI.S11093, PMID: 25114561 PMC4089835

[22] Barnes H Alexander S Bower L Ehlers J Gani C Herbert T . Development and results of a patient-reported treatment experience questionnaire on a 1.5 T MR-Linac. Clin Trans Radiat Oncol. (2021) 30:31–7. doi: 10.1016/j.ctro.2021.06.003, PMID: 34307911 PMC8283148

[23] Tetar S Bruynzeel A Bakker R Jeulink M Slotman BJ . Patient-reported outcome measurements on the tolerance of magnetic resonance imaging-guided radiation therapy. Cureus. (2018) 10., PMID: 29719739 10.7759/cureus.2236 PMC5922504

[24] Sayan M Serbez I Teymur B Gur G Zoto Mustafayev T Gungor G . Patient-reported tolerance of magnetic resonance-guided radiation therapy. Front Oncol. (2020) 10:1782. doi: 10.3389/fonc.2020.01782, PMID: 33072560 PMC7537416

[25] de Mol van Otterloo SR Westerhoff JM Leer T Rutgers RHA Meijers LTC Daamen LA . Patient expectation and experience of MR-guided radiotherapy using a 1.5T MR-Linac. Tech Innov Patient Support Radiat Oncol. (2024) 29:100224. doi: 10.1016/j.tipsro.2023.100224, PMID: 38162695 PMC10755768

[26] Moreira A Li W Berlin A Carpino-Rocca C Chung P Conroy L . Prospective evaluation of patient-reported anxiety and experiences with adaptive radiation therapy on an MR-linac. Tech Innov Patient Support Radiat Oncol. (2024) 29:100240. doi: 10.1016/j.tipsro.2024.100240, PMID: 38445180 PMC10912905

[27] Guckenberger M Baus WW Blanck O Combs SE Debus J Engenhart-Cabillic R . Definition and quality requirements for stereotactic radiotherapy: consensus statement from the DEGRO/DGMP Working Group Stereotactic Radiotherapy and Radiosurgery. Strahlentherapie und Onkologie. (2020) 196:417–20. doi: 10.1007/s00066-020-01603-1, PMID: 32211940 PMC7182610

[28] Klüter S Katayama S Spindeldreier CK Koerber SA Major G Alber M . First prospective clinical evaluation of feasibility and patient acceptance of magnetic resonance-guided radiotherapy in Germany. Strahlentherapie und Onkologie. (2020) 196:691–8. doi: 10.1007/s00066-020-01578-z, PMID: 32002567 PMC7385000

[29] Janssen TM Aitken K Alongi F Barry A Bernchou U Boeke S . First multicentre experience of SABR for lymph node and liver oligometastatic disease on the unity MR-Linac. Tech Innov Patient Support Radiat Oncol. (2022) 22:50–4. doi: 10.1016/j.tipsro.2022.04.005, PMID: 35586786 PMC9108982

[30] van Sörnsen de Koste JR Palacios MA Bruynzeel AME Slotman BJ Senan S Lagerwaard FJ . MR-guided gated stereotactic radiation therapy delivery for lung, adrenal, and pancreatic tumors: A geometric analysis. Int J Radiat Oncol Biol Physics. (2018) 102:858–66. doi: 10.1016/j.ijrobp.2018.05.048, PMID: 30061007

[31] Gani C Boeke S McNair H Ehlers J Nachbar M Mönnich D . Marker-less online MR-guided stereotactic body radiotherapy of liver metastases at a 1.5 T MR-Linac – Feasibility, workflow data and patient acceptance. Clin Trans Radiat Oncol. (2021) 26:55–61. doi: 10.1016/j.ctro.2020.11.014, PMID: 33319073 PMC7723999

[32] Uder L Nachbar M Butzer S Boldt J Baumeister S Bitzer M . Local control and patient reported outcomes after online MR guided stereotactic body radiotherapy of liver metastases. Front Oncol. (2023) 12:1095633. doi: 10.3389/fonc.2022.1095633, PMID: 36727060 PMC9885175

[33] Westerhoff JM Daamen LA Christodouleas JP Blezer EL Choudhury A Westley RL . Safety and tolerability of online adaptive high-field magnetic resonance–guided radiotherapy. JAMA network Open. (2024) 7:e2410819–e. doi: 10.1001/jamanetworkopen.2024.10819, PMID: 38691356 PMC11063805

[34] Jooya A Ly S Dawson L Moreira A Stanescu T Velec M . Patient-reported quality-of-life outcomes after abdominopelvic stereotactic body radiation therapy (SBRT) using an MR-linac system. Int J Radiat Oncol Biol Phys. (2025) 122:770–5. doi: 10.1016/j.ijrobp.2025.03.052, PMID: 40164350

[35] Barry AS Helou J Bezjak A Wong R Dawson LA Ringash J . Health related quality of life outcomes following stereotactic body radiotherapy in patients with oligo-metastatic disease: A systematic review and individual patient data meta-analysis. Radiotherapy Oncol. (2022) 173:163–9. doi: 10.1016/j.radonc.2022.05.033, PMID: 35680076

[36] Winter RM Boeke S Leibfarth S Habrich J Clasen K Nikolaou K . Clinical validation of a prognostic preclinical magnetic resonance imaging biomarker for radiotherapy outcome in head-and-neck cancer. Radiotherapy Oncol. (2025) 204:110702. doi: 10.1016/j.radonc.2024.110702, PMID: 39733969

[37] Boeke S Habrich J Kübler S Boldt J Schick F Nikolaou K . Longitudinal assessment of diffusion-weighted imaging during magnetic resonance-guided radiotherapy in head and neck cancer. Radiat Oncol (London England). (2025) 20:15. doi: 10.1186/s13014-025-02589-9, PMID: 39881423 PMC11780986

[38] Kishan AU Ma TM Lamb JM Casado M Wilhalme H Low DA . Magnetic resonance imaging–guided vs computed tomography–guided stereotactic body radiotherapy for prostate cancer: the MIRAGE randomized clinical trial. JAMA Oncol. (2023) 9:365–73. doi: 10.1001/jamaoncol.2022.6558, PMID: 36633877 PMC9857817

[39] Mannerberg A Persson E Jonsson J Gustafsson CJ Gunnlaugsson A Olsson LE . Dosimetric effects of adaptive prostate cancer radiotherapy in an MR-linac workflow. Radiation Oncology. (2020) 15:168. doi: 10.1186/s13014-020-01604-5, PMID: 32650811 PMC7350593

[40] Ocanto A Torres L Montijano M Rincón D Fernández C Sevilla B . MR-LINAC, a new partner in radiation oncology: current landscape. Cancers. (2024) 16:270. doi: 10.3390/cancers16020270, PMID: 38254760 PMC10813892

[41] Shimizuguchi T Nihei K Okano T Machitori Y Ito K Karasawa K . A comparison of clinical outcomes between three-dimensional conformal radiotherapy and intensity-modulated radiotherapy for prostate cancer. JIjoco. (2017) 22:373–9. doi: 10.1007/s10147-016-1057-y, PMID: 27778117

[42] Nutting CM Morden JP Harrington KJ Urbano TG Bhide SA Clark C . Parotid-sparing intensity modulated versus conventional radiotherapy in head and neck cancer (PARSPORT): a phase 3 multicentre randomised controlled trial. The Lancet Oncology. (2011) 12:127–36. doi: 10.1016/S1470-2045(10)70290-4, PMID: 21236730 PMC3033533

[43] Bradley JD Hu C Komaki RR Masters GA Blumenschein GR Schild SE . Long-term results of NRG oncology RTOG 0617: standard-versus high-dose chemoradiotherapy with or without cetuximab for unresectable stage III non–small-cell lung cancer. Journal of Clinical Oncology. (2020) 38:706–14. doi: 10.1200/JCO.19.01162, PMID: 31841363 PMC7048161

[44] Psoroulas S Paunoiu A Corradini S Hörner-Rieber J Tanadini-Lang S . MR-linac: role of artificial intelligence and automation. Strahlentherapie und Onkologie. (2025) 201:1–8. doi: 10.1007/s00066-024-02358-9, PMID: 39843783 PMC11839841

[45] Sibolt P Andersson LM Calmels L Sjöström D Bjelkengren U Geertsen P . Clinical implementation of artificial intelligence-driven cone-beam computed tomography-guided online adaptive radiotherapy in the pelvic region. Phys Imaging Radiat Oncol. (2021) 17:1–7. doi: 10.1016/j.phro.2020.12.004, PMID: 33898770 PMC8057957

[46] Kim J-Y Tawk B Knoll M Hoegen-Saßmannshausen P Liermann J Huber PE . Clinical workflow of cone beam computer tomography-based daily online adaptive radiotherapy with offline magnetic resonance guidance: the modular adaptive radiotherapy system (MARS). Cancers. (2024) 16:1210. doi: 10.3390/cancers16061210, PMID: 38539544 PMC10969008

[47] Weykamp F Schlemmer H-P Jäkel O Debus J . Combining CT-based online adaptive radiotherapy with offline MR guidance: the modular adaptive radiotherapy system (MARS). Cancers (2024). 16:1210. doi: 10.3390/cancers16061210, PMID: 38539544 PMC10969008

[48] Weykamp F Meixner E Arians N Hoegen-Saßmannshausen P Kim J-Y Tawk B . Daily AI-based treatment adaptation under weekly offline MR guidance in chemoradiotherapy for cervical cancer 1: the AIM-C1 trial. J Clin Med. (2024) 13:957. doi: 10.3390/jcm13040957, PMID: 38398270 PMC10889253

